# *In Vitro* Selection of a Single-Stranded DNA Molecular Recognition Element Specific for Bromacil

**DOI:** 10.1155/2014/102968

**Published:** 2014-10-23

**Authors:** Ryan M. Williams, Amanda R. Kulick, Srilakshmi Yedlapalli, Louisa Battistella, Cyrus J. Hajiran, Letha J. Sooter

**Affiliations:** ^1^Department of Basic Pharmaceutical Sciences, West Virginia University, 1 Medical Center Drive, P.O. Box 9530, Morgantown, WV 26506, USA; ^2^Memorial Sloan Kettering Cancer Center, Molecular Pharmacology & Chemistry Program, 1275 York Avenue, New York, NY 10065, USA; ^3^Department of Chemistry, West Virginia University, 217 Clark Hall, Morgantown, WV 26506, USA

## Abstract

Bromacil is a widely used herbicide that is known to contaminate environmental systems. Due to the hazards it presents and inefficient detection methods, it is necessary to create a rapid and efficient sensing device. Towards this end, we have utilized a stringent *in vitro* selection method to identify single-stranded DNA molecular recognition elements (MRE) specific for bromacil. We have identified one MRE with high affinity (*K*_*d*_ = 9.6 nM) and specificity for bromacil compared to negative targets of selection and other pesticides. The selected ssDNA MRE will be useful as the sensing element in a field-deployable bromacil detection device.

## 1. Introduction

Bromacil is a widely used herbicide in the substituted uracil family that is commercially used in the United States for annual and perennial brush and weed control in citrus and pineapple crops as well as in nonagricultural settings [1]. It is available under trade names such as Hyvar and Krovar and is popular due to a long soil residence of more than a year [2]. Between 55,000 and 117,000 pounds of bromacil were used on California citrus crops from 1992 to 2001 [3]. The positive economic impacts of its widespread use have led to many years of continuous environmental exposure to bromacil.

As a result of this widespread use, bromacil is a potentially hazardous environmental contaminant. It is known that bromacil is highly mobile in soils and can appear in ground waters following surface treatments, dependent upon soil composition and precipitation [4, 5]. This herbicide has been detected in groundwater wells and aquatic habitats at levels up to 5 *μ*g/L [5–7]. With these high levels of environmental contamination, it is likely that both humans and other plants and animals will be exposed to bromacil. In fact, it has been shown that dairy milk from cows that consume bromacil-treated food has relatively high bromacil levels [8].

These levels of bromacil in natural systems have clear environmental and human health consequences. The growth inhibition EC50 of bromacil for various aquatic algae, plant, and invertebrate animal species ranges from 5 to 500 *μ*g/L [7, 9]. Additionally, bromacil is a known toxin and developmental toxin to mice, rats, dogs, and sheep upon consumption and is a skin and mucosal irritant [10, 11]. Furthermore, bromacil is known to inhibit growth and nutrient uptake of human cell lines, be relatively genotoxic, and have carcinogenic potential [12–14]. Thus, bromacil is classified as a possible carcinogen by the US Environmental Protection Agency [9].

High levels of contamination and hazard risk caused the European Union to withdraw all commercial products containing bromacil in 2002 [15]. In spite of this, it is still found in soil and ground water throughout the EU. In the USA, the Environmental Protection Agency has set limits of 90 *μ*g/mL, 0.1 mg/kg/day, and 5000 *μ*g/L for lifetime health advisory, reference dose, and drinking water equivalent level, respectively [16]. Thus, it is important to monitor human and environmental exposure to bromacil.

Currently, however, detection of bromacil is costly, time-consuming, and labor-intensive. Chromatographic methods (gas and high pressure liquid) are historically used for bromacil detection and quantification in complex matrices [17]. There also have been mass spectrometry methods developed for bromacil and metabolite detection [15]. These require extensive sample preparation, expensive equipment, and expert operation. There have also been antibody-based enzyme-linked immunosorbent assays (ELISA) developed for bromacil detection [18–20]. Antibody-based assays, however, often suffer from lack of reusability, cost of use and production, and time to obtain results as well as inherent lack of specificity in typical antibody production [21]. Therefore, it is necessary to create a reusable, cost-efficient, rapid, and bromacil-specific binding element. This is best achieved through the selection of a stable single-stranded DNA (ssDNA) molecular recognition element (MRE).

The systematic evolution of ligands by exponential enrichment (SELEX) is a powerful method of obtaining such a molecule [22]. This iterative* in vitro* selection process enriches a library of up to 10^15^ different molecules for ones that bind to a target of interest with high affinity. The selected MRE is then useful in applications such as in target molecule sensing.

In this work, a stringent variation of the SELEX process was utilized. This methodology enriches the library for the target of selection as in conventional SELEX; however it places the greatest emphasis on what the MRE should not bind to. Multiple negative targets are utilized in the selection and are chosen by similarity of their chemical structure to the target molecule. Here, we have described the selection of a ssDNA MRE that binds with high affinity to the herbicide bromacil. We conducted highly stringent negative selections against the immobilization substrate and two closely related molecules: 6-amino-3-ethyl methyl uracil and 1-methyl uracil. Through this stringent SELEX process, we have identified a MRE with strong affinity and specificity for bromacil that may be incorporated in an environmental sensing device (e.g., [23, 24]).

## 2. Materials and Methods

### 2.1. *In Vitro* Selection of Bromacil-Specific MREs

**Figure 1:**
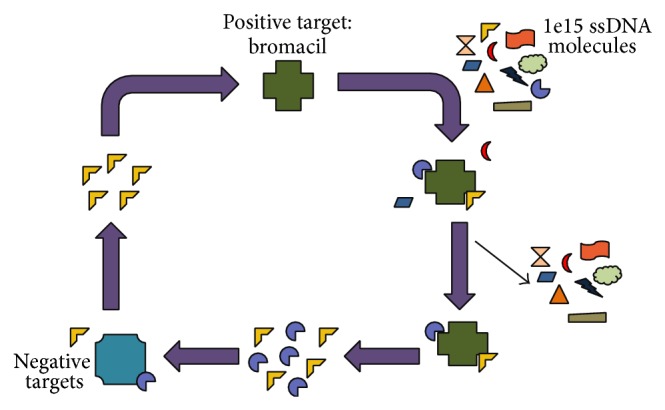
Scheme depicting the SELEX process. The target bromacil is incubated with a library of 10^15^ssDNA molecules. Those that bind are amplified and incubated with multiple negative targets. Those that do not bind the negative targets are amplified and subjected to additional rounds of* in vitro* selection.

A total of 13 rounds of SELEX were performed to obtain a bromacil-specific MRE (Figure 1). The initial library, which was composed of approximately 10^15^ molecules, was designed in our laboratory and termed RMW.N34 and was commercially synthesized (Eurofins MWG Operon; Huntsville, AL). It consisted of two 23 base constant regions flanking a 34-base random region. The library was amplified for those molecules that bound to bromacil and not closely related targets (Table 1).

**Figure 2:**
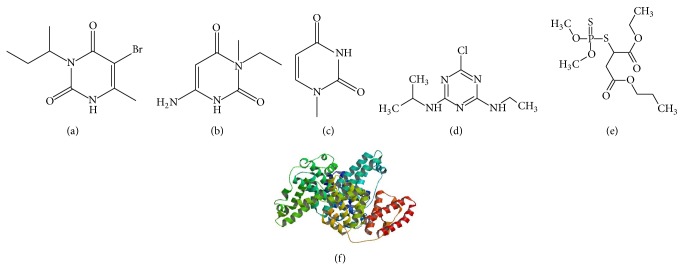
Structures of molecules used in the SELEX scheme and cross binding assays. (a) Structure of the herbicide and target of selection bromacil. (b) Structure of the bromacil metabolite 6-amino-3-ethyl methyl uracil, the 1st negative target. (c) Structure of the bromacil metabolite 1-methyl uracil, the 2nd negative target. (d) Structure of the herbicide atrazine, used in cross-reactivity binding assays. (e) Structure of the pesticide malathion, used in cross-reactivity binding assays. (f) Ribbon structure of bovine serum albumin (BSA) used in cross binding assays [25].

Bromacil (Figure 2(a)) (Sigma Aldrich; St. Louis, MO) was dissolved in 10% methanol and then covalently biotinylated with excess Sulfo-NHS-LC-Biotin (Pierce; Rockford, IL) according to manufacturer's protocol. Biotinylated bromocil was washed to remove excess biotin reagent and then conjugated to streptavidin-coated magnetic beads at a concentration of 1 nmol per 1 mg of magnetic beads given maximum binding (New England Biolabs; Ipswich, MA). Unbound biotinylated bromacil was washed to generate the immobilized target (IT) for selection.

For the first round of selection against bromacil [Round 1(+)], 50 *μ*L (200 pmol biotinylated bromacil bound to 200 *μ*g magnetic beads) of IT was incubated with the ssDNA in 500 *μ*L of 1X selection buffer (SB) composed of 100 mM sodium chloride, 20 mM Tris-HCl, and 2 mM magnesium chloride. The mixture was incubated with rotation for 48 hours at room temperature. Following incubation, unbound ssDNA was removed by magnetic separation and washed three times with 500 *μ*L SB. The IT with bound ssDNA was resuspended in 100 *μ*L SB, which served as template for polymerase chain reaction (PCR) amplification. Amplification ingredients consisted of enriched ssDNA library, 400 nM forward and biotinylated reverse RMW.N34 primers (Eurofins MWG Operon; Huntsville, AL) (forward: 5′-TGTACCGTCTGAGCGATTCGTAC-3′, biotinylated reverse: 5′-Biotin-GCACTCCTTAACACTGACTGGCT-3′), 250 *μ*M deoxynucleotide triphosphates, 1X GoTaq Reaction Buffer (Promega; Madison, WI), 3.5 units* Taq*, and pure water. Thermal cycling conditions were initial denaturation of 95°C for 5 minutes; cycling at 95°C for 1 minute, 63°C for 45 seconds, and 72°C for 1 minute; and final extension of 72°C for 7 minutes. Large-scale (3 mL) reactions were then performed for exponential amplification. This method of bromacil-targeted positive selection occurred through Round 7 with decreasing incubation times each round.

Following PCR amplification, dsDNA was purified with IBI DNA Fragment Extraction Kit (IBI Scientific; Peosta, IA). Eluted dsDNA containing a biotinylated reverse strand was then incubated with streptavidin agarose resin (Pierce; Rockford, IL) for strand separation. This was transferred into a flow-through column, washed with five volumes of phosphate-buffered saline, and five volumes of 1 M sodium hydroxide were then added to the column to elute the forward strand of ssDNA. Eluted ssDNA was purified by ethanol precipitation and resuspended in 50 *μ*L of SB. A NanoDrop spectrometer (Thermo Scientific; Rockford, IL) was used to confirm at least 10^13^ copies of ssDNA were present before proceeding to next round of selection.

The first round of negative selection [Round 1(−)] was performed by incubating Round 1(+) enriched ssDNA with 50 *μ*L (no bromacil and 200 *μ*g magnetic beads) immobilization substrate (IS) in a total volume of 100 *μ*L SB at room temperature for 24 hours with rotation. IS was prepared by blocking Sulfo-NHS-LC-Biotin with Tris-HCl and incubation with streptavidin-coated magnetic beads. After magnetic separation obtained supernatant with unbound ssDNA, washes with 25, 25, and 50 *μ*L SB were combined to serve as template for PCR amplification and were prepared for the next round of selection as noted above. Rounds 1–4 and 12 of negative selection were performed in this manner to obtain MREs specific for bromacil and not the IS.

Rounds 5(−), 6(−), and 7(−) selection were performed to select MREs which did not bind to 6-amino-3-ethyl methyl uracil (Figure 2(b)). This negative target was prepared in the same manner as IT (with identical volumes and concentrations), substituting bromacil with 6-amino-3-ethyl methyl uracil, to obtain the 1st immobilized negative target (INT-1). 50 *μ*L of INT-1 was incubated with enriched ssDNA from Round 5(+) in a total of 500 *μ*L SB at room temperature for 19 hours. Unbound ssDNA was removed with magnetic separation and served as template for PCR amplification and was prepared for the next round of selection as noted.

Beginning with Round 8(+), the selection was performed as noted above. However, after three washes, a competitive elution (CE) was performed by incubating the ssDNA bound to IT with 1 *μ*M free bromacil for 5 seconds. This was to ensure that MREs could bind well to free bromacil in solution. Supernatant, or ssDNA bound to free bromacil, was obtained by magnetic separation and served as the template for PCR amplification and was prepared for the next round of selection. This competitive elution was performed for positive rounds until Round 13(+), with decreasing concentrations, until finally 0.05 *μ*M was used.

Round 8(−) was performed identically to Round 5(−) selection, with the exception that the 2nd immobilized negative target (INT-2), 1-methyl uracil, was used (Figure 2(c)). A CE selection was performed for Round 9(−) and 10(−), identical to positive selection Rounds 8–13, with the 1st negative target 6-amino-3-ethyl methyl uracil and 2nd negative target 1-methyl uracil used instead of bromacil, respectively, to ensure the selected MREs did not bind to soluble negative targets. Round 11(−) CE selection was performed with the two negative targets in succession. ssDNA bound to the IT and not to free negative target was magnetically separated, suspended in 100 *μ*L SB, and PCR amplified for use in the following round of selection.

### 2.2. Sequencing of Library Diversity

Following every third round of selection until Round 12, and following Round 13(+), at least 30 random ssDNA sequences representative of the enriched library were obtained. Nonbiotinylated primers were used to obtain PCR-amplified enriched library and were ligated into the pCR2.1 vector (Invitrogen; Carlsbad, CA) following manufacturer's instructions. Ligated plasmid was cloned into chemically competent bacteria, amplified, and extracted using the AxyPrep Plasmid Prep Kit (Axygen; Union City, CA). Plasmids were sent for sequencing (Eurofins MWG Operon) using the M13R primer.

### 2.3. Bromacil MRE Binding Analysis

The Round 13(+) enriched ssDNA library sequences were analyzed for consensus binding sequences. Two of those, R13.2 and R13.26, were chosen for further analysis based on their structure, stability, and inclusion in consensus sequence families. The chosen sequences were characterized by the Mfold DNA web server using buffer salt conditions and 25°C [26–28].

Fluorescent saturation binding assays were performed to determine the binding affinity of R13.2 and R13.26 for bromacil essentially as previously described [29–31]. Concentrations of 0, 250, 500, 750, 1000, 50000, 100000, 200000, and 250000 pM of AlexaFluor 488 labeled MRE (Eurofins MWG Operon) were used in affinity binding studies. In a final volume of 200 *μ*L SB, 10 *μ*L IT was incubated with each concentration above for 5 minutes. Unbound MRE was removed by magnetic separation and washing five times each with 200 *μ*L SB. The IT was then resuspended in 200 *μ*L SB and the ssDNA was denatured by heating to 95°C for ten minutes. Eluted ssDNA was removed and fluorescence emission from the supernatant was measured by a Synergy 2 microplate reader with excitation at 490 nm from a tungsten halogen lamp and emission filter at 520 nm (BioTek US; Winooski, VT). Fluorescence measurements were normalized to a 100 *μ*L 1 nM solution in SB of the fluorescent MRE and background fluorescence was subtracted. For each concentration, the same was done with IS to ensure binding over background. Each set was performed in triplicate and the data averaged. The dissociation constant of the MREs was obtained in Origin 8 (OriginLab Corporation; Northampton, MA) using nonlinear regression analysis as previously described with the equation *Y* = ((Bmax∗*X*)/(*K*_*d*_ + *X*)) + NS∗*X* where Bmax is maximum binding, *K*_*d*_ is the dissociation constant, and NS is nonspecific binding [32].

To determine the cross-reactivity binding of the selected MREs, 100 nM fluorescently labeled ssDNA was incubated with IT and washed five times as noted above. Then, however, the ssDNA bound to IT was incubated with 1 *μ*M of the following in 0.01% methanol: bromacil as a positive control; negative targets 6-amino-3-ethyl methyl uracil and 1-methyl uracil; bovine serum albumin (BSA) as a model of a large, globular protein (Figure 2(f)) [33]; atrazine and malathion as examples of other pesticides found in similar environmental locations (Figures 2(d) and 2(e)) [34–36]; and SB. The supernatant, or ssDNA bound to free eluent, was magnetically removed and the fluorescence was measured as noted above. Each set was performed in triplicate, data were averaged, and standard deviations were calculated. For each eluent, a one-tailed *t*-test was performed to determine the statistical significance in difference of the means.

## 3. Results and Discussion

### 3.1. Identification of a Bromacil MRE

**Figure 3:**
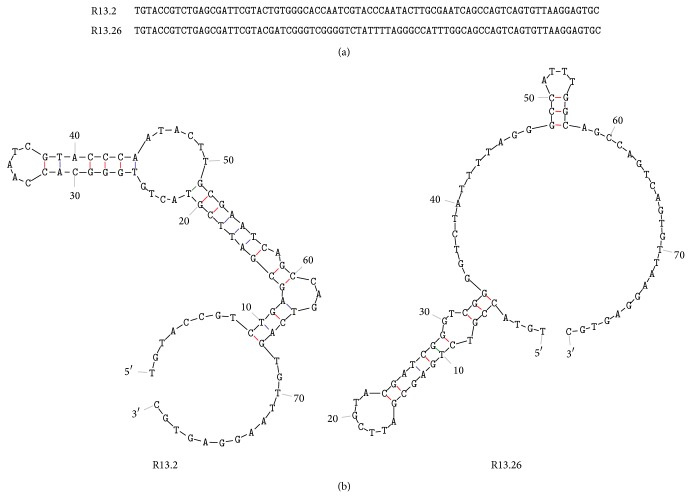
Sequence and structure of selected bromacil MREs. (a) ssDNA sequence of bromacil MREs R13.2 and R13.26 selected after 13 rounds of SELEX. (b) R13.2 and R13.26 secondary structure as predicted by Mfold [26, 27].

Thirteen rounds of SELEX were performed to identify a MRE with high affinity and specificity for the herbicide bromacil (Table 1). Negative selections were performed against bromacil metabolites 6-amino-3-ethyl methyl uracil and 1-methyl uracil as well as the immobilization substrate to ensure specificity. Following Round 13, two sequences were chosen from more than 30 based on inclusion in consensus sequence families, structure, and stability as predicted by the Mfold DNA web server (Table 2). These sequences, R13.2 and R13.26, have a predicted Gibb's free energy value of −8.32 and −9.64 kcal/mol, respectively (Figure 3).

### 3.2. Affinity and Specificity of Bromacil MRE

Fluorescence saturation binding assays identified R13.2 as having the highest affinity and specificity for bromacil, while R13.26 bound nonspecifically to magnetic beads with no bromacil and was therefore not further studied. We hypothesize that the binding motif of R13.2 is the GGGCA sequence that comprises a large sequence family (Table 2) and is present on the stem of a stem-loop structure (Figure 3). It should be noted that the GGGCA sequence also comprises a stem-loop structure found in R13.26 (Figure 3). Thus, it is possible that this structure is responsible for bromacil binding and other structures are responsible for the nonspecificity of R13.26. This sequence is present in three Round 13 sequences, one sequence in Round 9, four sequences in Round 6, and two sequences in Round 3. Thus, this sequence was selected early in the SELEX process and was present in high levels at the end of the selection.

**Figure 4:**
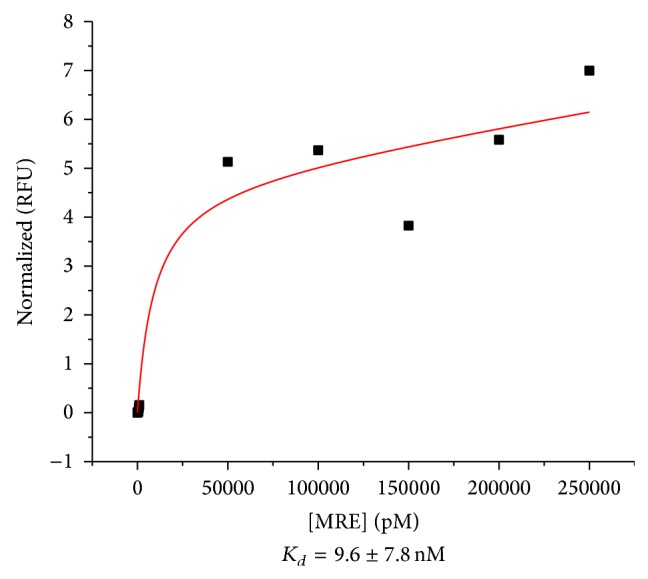
Affinity binding assays of R13.2. Top is a representative saturation binding curve of R13.2 with nonlinear regression best fit. Bottom is the equilibrium dissociation constant (*K*_*d*_) of R13.2 and bromacil (average of three experiments ± standard deviation).

The dissociation constant (*K*_*d*_) of R13.2 obtained from three fluorescence saturation binding assays was determined to be 9.6 ± 7.8 nM (Figure 4). This dissociation constant denotes a strong affinity of the MRE for bromacil, thus validating the stringency of the SELEX process. It is also within the lower range of affinities of MREs to other small molecule targets [37].

The cross-reactivity of R13.2 for the two metabolite negative targets was determined. The ssDNA MRE binds to bromacil more strongly than both metabolites selected against (Table 3). The binding of R13.2 to bromacil is 2.3 times greater than it is to the metabolite 6-amino 3-ethyl methyl uracil (*P* = 0.06) and 1.8 times greater than it is to 1-methyl uracil (*P* = 0.05). While binding of the MRE is not significantly greater for bromacil than 6-amino-3-ethyl methyl uracil, it is trending upon statistical significance. The lack of significance is due to large variation, which is likely caused by the strong likeness of the structure to bromacil. The selectivity of the MRE, however, is clear for bromacil over this metabolite. Thus, the ability of this stringent SELEX process to identify MREs that differentiate very closely related molecules is validated.

Furthermore, we determined the cross-reactivity of R13.2 for two other pesticides and bovine serum albumin (Table 3). The ssDNA binds to bromacil 2.6 times greater than BSA (*P* = 0.02), 2.3 times greater than atrazine (*P* = 0.04), and 5.2 times greater than malathion (*P* = 0.02). Additionally, nonspecific removal of the ssDNA MRE from immobilized bromacil by 0.01% methanol in SB is negligible (*P* = 0.006). Binding to BSA was determined to mimic binding to many other large, globular proteins [33] whereas atrazine and malathion are two pesticides found in similar environmental locations to bromacil [34–36]. This data suggests that R13.2 will be useful as the binding element in an environmental bromacil sensor.

The selected ssDNA MRE may be incorporated into a device which will allow rapid, portable, cost-efficient, and reusable monitoring of bromacil. Nucleic acid MREs for other targets have been used in such a capacity [38, 39]. For example, previous work has utilized MREs as the analyte capture element in microchip-based sensing devices, electrophoresis-based devices, and enzyme-linked immunosorbent assays (ELISAs) [40–42]. Potential drawbacks associated with ssDNA MRE environmental sensing include nuclease degradation of the sensing element, sensitivity, and specificity compared to conventional methods [43]. However, the potential advantages of a ssDNA MRE device are noteworthy when compared to existing bromacil detection methods, such as chromatography, mass spectrometry, and ELISAs with antibodies. Future work will focus on the development of a sensor incorporating this MRE. With greater detection of bromacil contamination, it will be possible to rapidly warn the public of contaminated water sources and possibly remediate these contaminated environments by bacterial degradation, biofiltration, reverse osmosis, or other methods [44, 45]. This will lead to a reduction of environmental contamination and related consequences from widespread bromacil use.

## 4. Conclusions

A stringent* in vitro *selection variation has been developed and applied for the identification of a bromacil-specific molecular recognition element. Thirteen rounds of selection with strong stringency for bromacil and against closely related metabolites and the immobilization substrate were performed. The selected ssDNA MRE has a high affinity and strong selectivity for bromacil. This work has developed and validated a stringent method for the selection of specific MREs and obtained a ssDNA MRE for the herbicide bromacil, which will lead to monitoring and potential remediation of contaminated environments.

## Figures and Tables

**Table 1:** SELEX scheme for selection of a bromacil-specific MRE.

Round	Positive selection	Negative selection
1	Immobilized target (IT) 48 hrs	Immobilization substrate (IS) 24 hrs
2	IT 18 hrs	IS 23 hrs
3	IT 12 hrs	IS 12 hrs
4	IT 6 hrs	IS 6 hrs
5	IT 1 hrs	6-Amino-3-ethyl methyl uracil (1st immobilized negative target INT-1) 19 hrs
6	IT 5 min	INT-1 19 hrs
7	IT 5 sec	INT-1 18 hrs
8	IT 5 sec, 1 *μ*M bromacil competitive elution (CE)	1-Methyl uracil (2nd immobilized negative target INT-2)
9	IT 5 sec, 0.5 *μ*M bromacil CE	IT 5 sec, CE with NT-1 5 sec
10	IT 5 sec, 0.1 *μ*M bromacil CE	IT 5 sec, CE with NT-2 5 sec
11	IT 5 sec, 0.05 *μ*M bromacil CE	IT 5 sec, CE with NT-1 5 sec, CE with NT-2 5 sec
12	IT 5 sec, 0.01 *μ*M bromacil CE	IS 24 hrs
13	IT 5 sec, 0.05 *μ*M bromacil CE	

*In vitro* selection process for obtaining a bromacil-specific MRE. Immobilization target (IT) is bromacil bound to magnet beads. Immobilization substrate (IS) is streptavidin-coated magnetic beads plus blocked biotin reagent. Competitive elution (CE) is removal of bound ssDNA from target-coated magnetic beads by free pesticide in solution. INT-1 or NT-1 is the 1st immobilized or free negative target 6-amino-3-ethyl methyl uracil. INT-2 or NT-2 is the 2nd immobilized or free negative target 1-methyl uracil. Times listed are incubation times in hours (hrs), minutes (min), or seconds (sec).

**Table 2:** R13 sequence families.

Name	Sequence
R 13.2	TGTACCGTCTGAGCGATTCGTACTGTGGGCACCAATCGTACCCAATACTTGCGAATCAGCCAGTCAGTGTTAAGGAGTGC
R13.4	TGTACCGTCTGAGCGATTCGTACCCCGCCCGTAGTTGACTGAGCTAGGTGGGCCTGAAGCCAGTCAGTGTTAAGGAGTGC
R13.10	TGTACCGTCTGAGCGATTCGTACTGTTTCTTAGAGGGTTTTTGGGCTGCAAATCAGAAGCCAGTCAGTGTTAAGGAGTGC
R13.14	TGTACCGTCTGAGCGATTCGTACGATCGGGTCGGGGTCTATTTTAGGGCCATTTGGCAGCCAGTCAGTGTTAAGGAGTGC
R13.26	TGTACCGTCTGAGCGATTCGTACGATCGGGTCGGGGTCTATTTTAGGGCCATTTGGCAGCCAGTCAGTGTTAAGGAGTGC
R13.1	TGTACCGTCTGAGCGATTCGTACCAACAACGGTGCTTTGAGGGTTACAGGGCAGCTGAGCCAGTCAGTGTTAAGGAGTGC
R13.34	TGTACCGTCTGAGCGATTCGTACACTAGTGCGAGGTGCCTATCTACAGTCGGGGCATAGCCAGTCAGTGTTAAGGAGTGC

R13.2	TGTACCGTCTGAGCGATTCGTACTGTGGGCACCAATCGTACCCAATACTTGCGAATCAGCCAGTCAGTGTTAAGGAGTGC
R13.10	TGTACCGTCTGAGCGATTCGTACTGTTTCTTAGAGGGTTTTTGGGCTGCAAATCAGAAGCCAGTCAGTGTTAAGGAGTGC
R13.20	TGTACCGTCTGAGCGATTCGTACGGCTGCGGACATCAGCAGAGAAGTATCCGGTGATAGCCAGTCAGTGTTAAGGAGTGC
R13.24	TGTACCGTCTGAGCGATTCGTACGGTTAGAACGGGGGCCGGGGCTGCTCGGTATATCAGCCAGTCAGTGTTAAGGAGTGC
R13.16	TGTACCGTCTGAGCGATTCGTACGGCCGGAGAGTGTCGTATCAGCAGATTAGCGAGGAGCCAGTCAGTGTTAAGGAGTGC

R13.26	TGTACCGTCTGAGCGATTCGTACGATCGGGTCGGGGTCTATTTTAGGGCCATTTGGCAGCCAGTCAGTGTTAAGGAGTGC
R13.34	TGTACCGTCTGAGCGATTCGTACACTAGTGCGAGGTGCCTATCTACAGTCGGGGCATAGCCAGTCAGTGTTAAGGAGTGC
R13.6	TGTACCGTCTGAGCGATTCGTACTTGTGGGGTCGTACTAGTTCGCTTTTCTGGAGGTAGCCAGTCAGTGTTAAGGAGTGC
R13.24	TGTACCGTCTGAGCGATTCGTACGGTTAGAACGGGGGCCGGGGCTGCTCGGTATATCAGCCAGTCAGTGTTAAGGAGTGC
R13.24	TGTACCGTCTGAGCGATTCGTACGGTTAGAACGGGGGCCGGGGCTGCTCGGTATATCAGCCAGTCAGTGTTAAGGAGTGC

Representative sequence families following Round 13 of SELEX. Families are separated by horizontal line with common sequences underlined.

**Table 3:** Cross-reactivity data of R13.2 ssDNA MRE.

Eluent	Average fluorescence (RFU)	Standard deviation	*P* value	Selectivity ratio
Bromacil	0.51	0.16	—	—
NT-1	0.22	0.20	0.06	2.3
NT-2	0.28	0.09	0.05	1.8
BSA	0.19	0.08	0.02	2.6
Atrazine	0.22	0.15	0.04	2.3
Malathion	0.10	0.15	0.02	5.2
0.01% MeOH	−0.0045	0.12	0.006	—

For each eluent, average fluorescence in solution is given with standard deviation. The *P* value is given from a student's *t*-test performed between the eluent and bromacil. The selectivity ratio is the number of times greater binding to bromacil than to the eluent. All assays were performed in 0.01% methanol due to solubility; thus removal of R13.2 from bromacil was determined to be negligible.
